# Possible Role of Dopamine in the Enhancement of Intrahippocampal Arc Protein Expression Induced by Post-Learning Noradrenergic Stimulation of the Basolateral Amygdala

**DOI:** 10.3390/ijms27031273

**Published:** 2026-01-27

**Authors:** Bogomil Peshev, Dimitrinka Atanasova, Pavel Rashev, Lidia Kortenska, Milena Mourdjeva, Despina Pupaki, Nikolaos Efstratiou, Nikolay Dimitrov, Jana Tchekalarova

**Affiliations:** 1 Institute of Neurobiology, Bulgarian Academy of Sciences, 1113 Sofia, Bulgaria; bogpeshev@gmail.com (B.P.); didiatanasova7@gmail.com (D.A.);; 2 Department of Anatomy, Faculty of Medicine, Trakia University, 6003 Stara Zagora, Bulgaria; 3 Institute of Biology and Immunology of Reproduction, Bulgarian Academy of Sciences, 1113 Sofia, Bulgaria; pavel_rashev@abv.bg (P.R.);

**Keywords:** noradrenergic transmission, basolateral amygdala, consolidation, dopamine receptors, dorsal hippocampus

## Abstract

Extensive research in laboratory rodents has shown that novelty exposure enhances the consolidation of memories for preceding or following low-arousal events by elevating dopamine release in the dorsal hippocampus (dHipp). Additionally, numerous studies have demonstrated that post-encoding noradrenergic activation of the basolateral amygdala (BLA) can also enhance memory consolidation in dHipp. Since the BLA is most active during emotionally arousing or stress-related situations, it was suggested that this nuclear complex mediates the effects of emotional salience on memory consolidation. However, it is presently unknown whether the reinforcement of memory storage in dHipp induced by novel and arousing environmental conditions results from the interaction between these two modulatory systems. To test the hypothesis of a functional interaction between dopaminergic and noradrenergic systems, this study assessed their combined effects on a low-arousal object-location (OL) task. Rats received post-training intra-BLA infusions of vehicle or clenbuterol (Clen), a selective β-adrenoceptor agonist. Novelty-induced dopamine release in the dHipp was enhanced by omitting habituation prior to training, and the contribution of dopamine signaling was further evaluated using pre-infusion administration of the D1/D5 receptor antagonist SCH 23390. The distribution of two important proteins for memory processing, namely the activity-regulated cytoskeleton-associated protein (Arc) and the phosphorylated form of the transcription factor, cAMP-response element-binding protein (pCREB) in the dHipp, was explored in a subset of rats perfused 60 min after the training phase. Stimulation of the BLA significantly increased the number of Arc- and pCREB-positive cells in several dHipp areas. The preceding application of SCH 23390, however, substantially decreased these effects in the same areas, i.e., the dentate gyrus (DG), CA2, and CA1 subregions for pCREB, and the CA3b, CA3c, CA2, and CA1 subregions for Arc. This interaction is considered essential for the initial stages of memory consolidation. The obtained results indicate the presence of a region-specific interaction between BLA modulatory inputs and intrahippocampal dopaminergic transmission, the mechanisms of which remain to be elucidated.

## 1. Introduction

The ability of vertebrates to store information about environmental stimuli is essential to their adaptability. This capacity allows them to make memory-based predictions that guide their motivated behaviors in a constantly changing environment. In laboratory rodents, which are the most commonly used model organisms in research on learning and memory, the ability to form, preserve, and retrieve associations between stimuli and the specific spatiotemporal context in which they were experienced (episodic-like memory) is linked to the activation of a neuronal population in the dorsal regions of the hippocampus. (dHipp) [1,2,3]. The formation of memory traces for specific events and the future accessibility of stored information are determined by the ability of hippocampal neurons to adjust their connectivity by altering the properties of their synaptic contacts. The experience-driven change in synaptic strength, universally known as long-term potentiation (LTP), is initiated during memory encoding and requires functional and structural modifications, such as the integration of additional glutamatergic AMPA receptors in the postsynaptic membrane and actin-dependent reconfiguration of postsynaptic dendritic spines [4,5]. This initial stage is called early-LTP [6].

Nevertheless, the changes in cellular activity that lead to the establishment of early-LTP are not sufficient to fully ensure the long-term preservation of newly acquired information. According to one of the most influential models in present-day neurobiology of learning and memory, the synaptic tagging and capture hypothesis, for an early-LTP to completely mature and reach its long-lasting form (long-LTP), therefore transforming a malleable short-term memory into an enduring one, subsequent waves of protein synthesis are required [7,8]. Thus, while early-LTP induction during memory acquisition is protein synthesis-independent, the long-term stabilization of memories is supported by a separate maintenance phase of molecular and genomic interactions, which leads to de novo protein synthesis to sustain synaptic modifications and prepare the information-storing neurons for future reactivation. This later stage of memory formation is known as initial (cellular) memory consolidation.

However, not all events carry the same relevance for an individual’s well-being, and therefore, not every experience is capable of triggering the complex biological mechanisms required for early-LTP reinforcement. The dynamics of initial memory consolidation depend on the characteristics of the environment in which the experience occurs and the aspects of the encountered stimuli. Events characterized by novelty, unexpectedness, and/or emotional significance lead to the establishment of more stable memory traces in dHipp, as evidenced by improved performance during memory retention tests [9,10,11]. In the dHipp, salient experiences, such as unexpected encounters with aversive stimuli or exposure to a previously unexplored environmental setting, lead to upregulation of the catecholaminergic neuromodulators dopamine and noradrenaline [10,11]. When these signaling molecules bind to their respective metabotropic receptors (e.g., D1/D5 receptors, beta-adrenergic receptors), they activate intracellular signaling cascades that control the expression of genes indispensable for memory storage and the synthesis of proteins involved in synaptic plasticity. It has been shown that the administration of beta-adrenoreceptor antagonist propranolol prevents the transformation of tetanically induced early-LTP into long-LTP in the dentate gyrus (DG) region of the hippocampus in rats [9]. Furthermore, intracerebroventricular (icv) application of propranolol blocked the reinforcement effect of preceding novelty exposure on tetanically induced early-LTP in the DG. Similarly, it has been demonstrated that icv injection of the selective D1/D5 receptor antagonist, SCH 23390, prevents novelty-dependent facilitation of LTP induction in the CA1 area of the hippocampus [12]. Interestingly, the same study reports no effect of propranolol injection in this hippocampal region, suggesting regional specificity in the impact these neuromodulators have on experience-driven synaptic plasticity and memory consolidation in the hippocampus. Indeed, it has been previously shown, through both in vivo and in vitro experiments, that while dopamine plays a significant role in facilitating late-LTP in the CA1 region, noradrenaline has a more profound impact on synaptic strength modifications in the dentate gyrus (DG) [13]. While it was widely believed that the VTA was solely responsible for the dopaminergic modulation of memory processes, many recent behavioral studies have provided evidence that the primary source of noradrenaline in dHipp, the locus coreuleus (LC), is also capable of releasing dopamine under distinctively novel environmental conditions or after the surprising occurrence of a salient stimulus [14].

Besides the ventral tegmental area (VTA) and LC, in relation to emotionally salient and unpredictable events, other brain structures could also influence information storage in dHipp both directly and indirectly [11,15]. A distinct pivotal line of research, yet conceptually close to the work examining the role of novelty-induced catecholaminergic modulation of memory consolidation, has identified the basolateral amygdala (BLA) as a key modulatory node capable of influencing the dynamics of molecular processes responsible for synaptic strength modifications in dHipp [16]. The BLA is a set of interconnected nuclei located in the medial temporal lobe of the brain in mammalian species, part of the greater amygdaloid complex, which has been extensively studied in the context of emotional learning, encoding of the emotional valence for both rewarding and aversive stimuli, and control over motivated behaviors. Over the past few decades, numerous studies have demonstrated that pharmacological, electrophysiological, or optogenetic stimulation of the BLA immediately after learning enhances initial memory consolidation, resulting in more stable long-term memory [16,17,18]. These findings have led to the theoretical presumption that during emotionally arousing or stressful situations, the BLA plays a vital role in regulating the selectivity and quality of processes involved in memory formation. Moreover, it has been abundantly demonstrated that the positive effect of BLA activation on synaptic plasticity and memory consolidation in dHipp is dependent on noradrenergic transmission in the area [16,19]. Neuropharmacological studies have revealed that the post-training infusion of the selective beta-adrenoreceptor agonist clenbuterol (Clen) in the BLA increases the levels of activity-regulated cytoskeleton-associated protein (Arc) in the dHipp and enhances the subsequent memory retrieval [20,21,22]. Furthermore, the above-cited studies of the modulatory function of the BLA on memory consolidation have been conducted using a range of behavioral paradigms, from emotionally neutral spontaneous recognition tasks to low-intensity aversive or appetitive tasks. These results demonstrate that BLA activation can modulate the mechanism of memory storage in the hippocampus, regardless of the nature of the information to be preserved.

Considering the research outlined above, the present study set out to investigate a plausible interaction between noradrenergic activation of the BLA and dopaminergic neurotransmission in the dHipp in the modulation of initial memory consolidation. Understanding under what environmental conditions different modulatory systems are activated and how they interact when influencing neuronal activity and synaptic plasticity in dHipp will greatly impact existing theoretical concepts of memory consolidation and information storage. Our experimental design used a well-established scheme, combining the low-arousing hippocampal-dependent recognition memory protocol with the object-location (OL) task and post-training pharmacological stimulation of the BLA via direct infusion of Clen. Additionally, to test whether the BLA could influence initial memory consolidation when dopaminergic activity in dHipp is severely disrupted, a separate group of experimental animals received a systemic injection of D1/D5 receptor antagonist, SCH 23390. We conducted two separate experiments to examine both the anatomical dimensions and the behavioral effects of this hypothetical interaction. In our first experiment, a small number of animals from all experimental groups were sacrificed 60 min after pharmacological manipulations, and their brains were collected for immunohistochemical analysis. Similar to some of the abovementioned studies investigating the modulatory effects of the BLA on memory consolidation in dHipp [20,21,22], we also examined Arc as a marker of experience-driven protein expression and memory-related activity. However, unlike these studies, we focused on the anatomical distribution of the protein in the dHipp rather than on synapse-level expression. In doing so, we wanted to establish if noradrenergic stimulation of the BLA affects the entire dHipp and in which hippocampal regions Arc expression would be most susceptible to dopaminergic receptor blockade. Together with Arc, we also examined pCREB distribution, as this transcription factor is an important marker of neuronal excitability and novelty detection in dHipp. In our second experiment, the animals from all groups were returned to the experimental setup 24 h after training to assess the effect of the noradrenergic stimulation of the BLA on spatial recognition. After thorough analysis of the obtained immunohistochemical data, we concluded that the BLA may interact with intrahippocampal dopaminergic neuromodulation in a region-specific manner, influencing experience-induced plasticity mainly in the medial and distal CA3, CA2, and CA1 areas, respectively. While the BLA could also influence protein expression and information processing in the DG and the proximal CA3 area, it does not appear that an interaction with dopamine is required to affect these dHipp regions. To our surprise, Clen-induced BLA activation did not enhance the animals’ performance during the test session of the OL task. However, this lack of behavioral effect may be due to higher anxiety levels during the training session, as the animals were not habituated prior to the experiment. Nevertheless, we believe that the evidence for regionally specific relation with intrahippocampal dopamine neurotransmission reported here represents a valuable addition to our current understanding of BLA-dependent modulation of hippocampal activity and that future modifications of our behavioral protocols will allow us to study the conditions under which this functional relationship between these two modulatory systems in more depth.

## 2. Results

**Figure 1:**
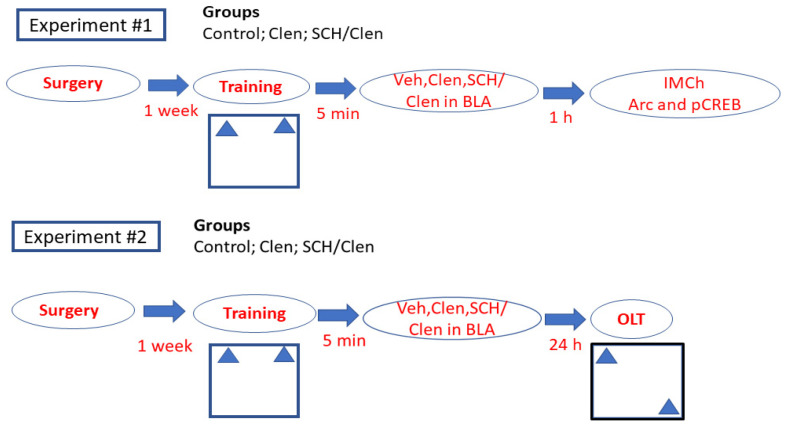
Experimental timeline. Abbreviations: clenbuterol (Clen); SCH 23390 (SCH); vehicle (Veh); basolateral amygdala (BLA); immunohistochemistry (IMCh); activity-regulated cytoskeleton-associated protein (Arc); object location test (OLT).

The experimental timeline is shown in Figure 1. One week after the surgery procedure, animals were randomly assigned to two cohorts of three experimental groups prior to behavioral testing. Group 1 (Control), Group 2 Clen, and Group 3 SCH 23390/Clen (SCH/Clen) were trained in the OL paradigm (training phase). The first cohort of rats was perfused for IMC 1 h after the training phase, while the second cohort was tested in the OL test 24 h later.

### 2.1. Experiment #1: Post-Training Systemic SCH 23390 Treatment Suppresses the Potentiating Effect of Clenbuterol (Clen) Infused into the Basolateral Amygdala (BLA) on Hippocampal pCREB and Arc Expression in the Dentate Gyrus (DG) and Cornu Ammonis

To evaluate the role of dopaminergic D1/D5 receptor activation on the noradrenergic-related modulation of the BLA on the pCREB and Arc protein expression in the dHipp, which is suggested to enhance the consolidation process, the three groups (Control, Clen, and SCH 23390 + Clen (SCH/Clen)) were euthanized 60 min after the sample test. Immunohistochemistry (IMC) was used to explore the expression of the phosphorylated form of the transcription factor and the plastic marker Arc in the granular dentatus gyrus (GrDG) and polymorphic layer of the DG (PoDG), as well as in the cornu ammonis (CA).

**Figure 2:**
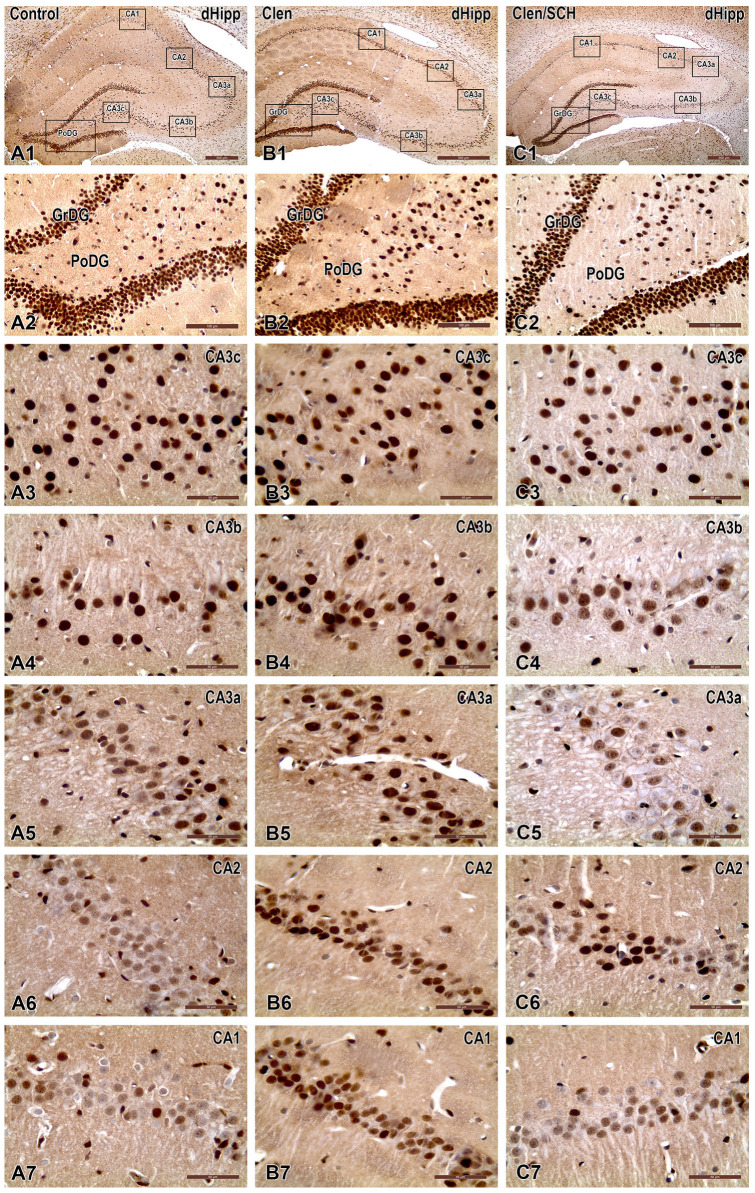
Representative immunohistochemistry for phosphorylated CREB (pCREB) in the dorsal hippocampus (dHipp) of rats, showing nuclear pCREB immunoreactivity (brown DAB; hematoxylin counterstain) in coronal sections from Control (A-panels), clenbuterol (Clen)-treated (Clen; B-panels), and SCH + Clen 23390 (SCH) co-treated (SCH/Clen; C-panels) groups. Low-magnification overviews (**A1**–**C1**) depict the dHipp with boxed sampling sites, while higher-magnification images illustrate the dentate gyrus ((**A2**–**C2**); granule cell layer, GrDG; polymorphic layer, PoDG), the CA3 subfields ((**A3**–**C3**,**A4**–**C4**,**A5**–**C5**) corresponding to CA3c, CA3b, and CA3a), CA2 (**A6**–**C6**), and CA1 (**A7**–**C7**). Compared to the Control, the Clen-treated group shows a visibly greater number and intensity of pCREB-positive nuclei across subfields—most pronounced in the GrDG and CA3/CA1 pyramidal layers, whereas co-administration with SCH attenuates this labeling toward control levels. Scale bars: 500 µm (**A1**–**C1**); 100 µm (**A2**–**C2**); 50 µm (**A3**–**C3**,**A4**–**C4**,**A5**–**C5**,**A6**–**C6**,**A7**–**C7**).

Staining for pCREB (phosphor-CREB Ser133) using the Affinity Biosciences antibody (Cat. AF3189, lot #1479) revealed a predominantly nuclear DAB reaction product in the DG and CA fields, with a light hematoxylin counterstaining the somata and cytoarchitecture (Figure 2).

**Figure 3:**
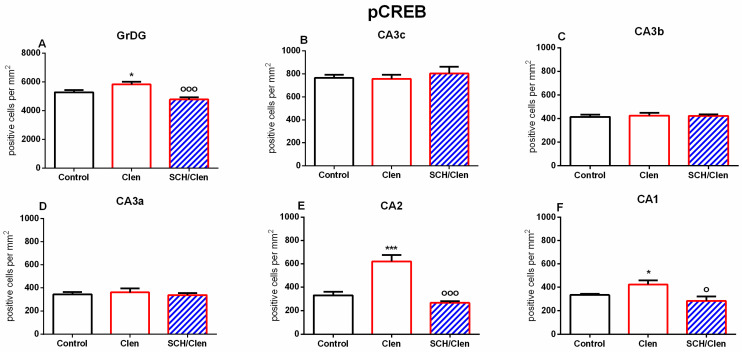
The density of pCREB-positive cells (cells/mm^2^) in various parts of the dHipp: GrDG (**A**), CA3c (**B**), CA3b (**C**), CA3a (**D**), CA2 (**E**), and CA1 (**F**). Three groups of animals are represented: Control (black), Clen-treated (red), and SCH/Clen co-treated (red with blue stripes). Administration of Clen led to a significant increase in the density of pCREB-positive cells in GrDG, CA2, and CA1 compared with Control. The co-treatment with SCH reversed these effects; the number of pCREB-positive cells in GrDG, CA2, and CA1 was significantly lowered. Kruskal–Wallis one-way ANOVA, H = 15.308, *p* < 0.001—GrDG; H = 20.042, *p* < 0.001—CA2; H = 20.532, *p* < 0.001—CA1. (Dunn’s post hoc test: * *p* < 0.05, *** *p* < 0.001 vs. Control; ° *p* < 0.05, °°° *p* < 0.001 vs. Clen). No statistically significant differences were detected in CA3 subfields (*p* > 0.05). The results are presented as mean ± SEM; n = 5 per group.

The quantification of pCREB-positive nuclei per mm^2^ is presented in Figure 2. In the GrDG, pCREB immunoreactivity increased slightly upon β_2_-adrenergic stimulation with Clen as compared to the Control group (*p* < 0.05) (Figure 3A). Co-administration of Clen and SCH 23390 (SCH/Clen) markedly reduced this effect (*p* < 0.001 vs. Clen), bringing levels of expression back in line with those in the Control group. There were no significant differences between groups across the CA3 subfields (CA3c, CA3b, and CA3a) (Figure 3B–D). The micrographs also show similar nuclear pCREB densities and intensities across all conditions (Figure 2(A3–A5,B3–B5,C3–C5)). In the CA2, Clen markedly increased the number of pCREB-positive nuclei (*p* < 0.001 vs. Control), whereas SCH abolished this increase (*p* < 0.001 vs. Clen) (Figure 3F). Representative images at both low and high magnification levels demonstrate neurochemical staining patterns that correspond to each subfield: more pronounced nuclear staining in DG, CA2, and CA1 after Clen; selective reversal by SCH in CA2 and CA1; and very few changes in CA3 (Figure 2). Taken together, these findings point to a region-dependent pCREB response to β_2_-adrenergic activation, with D1/D5 sensitive modulation in GrDG, CA2, and CA1.

**Figure 4:**
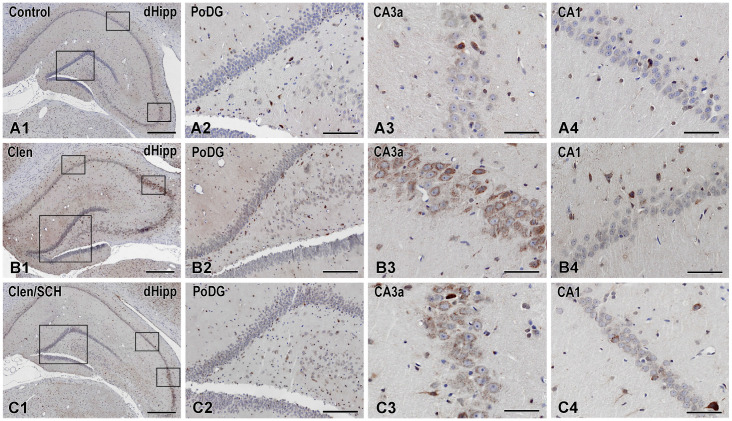
Representative Arc immunohistochemistry in the dHipp following BLA β_2_-adrenergic activation and subsequent antagonism by SCH 23390. Representative bright-field micrographs of Arc immunohistochemistry (DAB chromogen, brown) with hematoxylin counterstain (blue) in the dHipp. The rows show the experimental groups: control, Clen, and SCH/Clen. From left to right, the panels (**A1**–**C1**) depict a low-magnification overview of the dHipp with sampling frames, followed by higher-magnification views of subfields ((**A2**–**C2**) GrDG/PoDG, (**A3**–**C3**) CA3a, and (**A4**–**C4**) CA1). Clenbuterol increases the number of Arc-positive neurons in the CA fields (notably in CA3a and CA1), whereas in the GrDG/PoDG, no significant change is observed. Co-administration of SCH reduces the Clen-evoked Arc signal, normalizing CA1 and markedly attenuating CA3a. Scale bars: 500 µm (**A1**–**C1**); 200 µm (**A2**–**C2**); 100 µm (**A3**,**A4**,**B3**,**B4**,**C3**,**C4**).

Arc immunoreactivity is always found outside the nucleus, with granular to diffuse DAB deposits in the cytoplasm and proximal dendrites of neurons. The nuclei stained with hematoxylin stay blue, suggesting that there is no nuclear Arc labelling (Figure 4). Arc immunoreactivity in the dHipp was minimal and localized to a few cells in the CA areas and DG of control slices (Figure 4(A1–A4)). These results are consistent with the low tonic activity of a plasticity marker from the immediate early gene family. After infusion of the β_2_-adrenergic receptor agonist Clen into the BLA, Arc labelling changed significantly throughout the hippocampus. The most significant increase was found in CA3a and CA3b (Figure 4(B3)), followed by CA1 (Figure 4(B4)) and CA3c, all of which showed a visible increase in Arc labelling compared to the Control. Conversely, the GrDG and the hilar layer (PoDG) showed no significant change (Figure 4(B2)).

**Figure 5:**
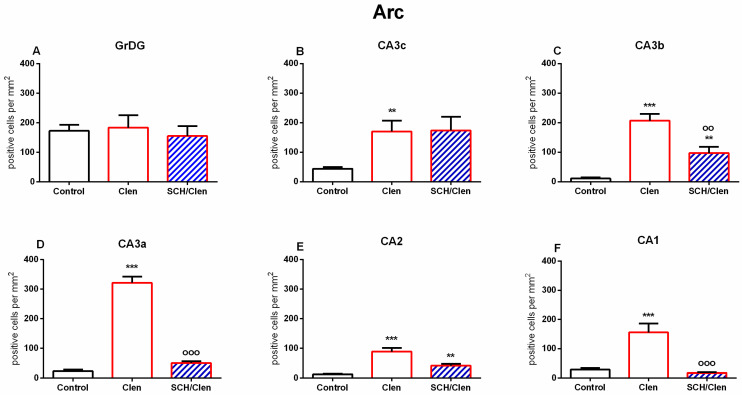
Quantification of Arc-positive cells in the DG and hippocampal subfields following treatment with clenbuterol and SCH 23390. Bar diagrams illustrate the density of Arc-positive cells (cells/mm^2^) in the following hippocampus subfields: GrDG (**A**), CA3c (**B**), CA3b (**C**), CA3a (**D**), CA2 (**E**), and CA1 (**F**). Three groups of experiments are presented: Control (white bars), Clen-treated (red bars), and SCH/Clen co-treated (red bars with blue diagonal stripes). Compared with the Control, Clen administration significantly increased the density of Arc-positive cells in CA3c, CA3b, CA3a, CA2, and CA1. SCH 23390 treatment combined with clenbuterol diminished the effects, thereby significantly reducing the levels of Arc-positive cells in CA3b, CA3a, CA2, and CA1. Kruskal–Wallis one-way ANOVA, H = 6663, *p* = 0.036—CA3c; H = 23,052, *p* < 0.001—CA3b; H = 50,064, *p* < 0.001—CA3a; H = 23,454, *p* < 0.001—CA2; H = 36,432, *p* < 0.001—CA1. (Dunn’s post hoc test: ** *p* < 0.01, *** *p* < 0.001 vs. control; °° *p* < 0.01, °°° *p* < 0.001 vs. Clen). No significant differences were detected between groups in the GrDG (*p* > 0.05). The data are presented as mean ± SEM; n = 5 per group.

Following Clen infusion in the BLA, an increased Arc expression specific to certain regions was observed in the dHipp. Thus, although the β_2_-adrenergic receptor agonist had no effect on Arc expression in the GrDG (Figure 4(A2–C2) and Figure 5A), it significantly increased the expression of this plasticity marker in the CA3c, CA3b, CA3a, CA2, and CA1 regions of the CA (*p* < 0.01, *p* < 0.001, for the Clen vs. Control group) (Figure 4(A3,A4,B3,B4,C3,C4) and Figure 5B–F). Pharmacological co-manipulation of dopaminergic signaling dramatically limited the Clen-induced changes in gene expression. The D1/D5 receptor antagonist SCH, when administered alongside the Clen tended to reduce Arc expression compared to the Clen alone in the CA3b region (*p* < 0.01 vs. Control and vs. Clen, respectively) (Figure 4(C1) and Figure 5C) as well as in the CA3a, CA2, and CA1 regions (*p* < 0.001 vs. Clen) (Figure 5D–F). This is evident in the corresponding figures as a substantial disappearance of parts of the CA3a (Figure 4(C3)) and CA1 (Figure 4(C4)) layers, where Arc-positive neurons were heavily stained in the SCH condition. In contrast, the Clen-alone sections show that those same strata are full of frequently and strongly DAB-positive neurons. As shown in the figures and confirmed by the statistics, there was no significant difference in Arc expression between the treatment groups in GrDG (Figure 5A).

**Figure 6:**
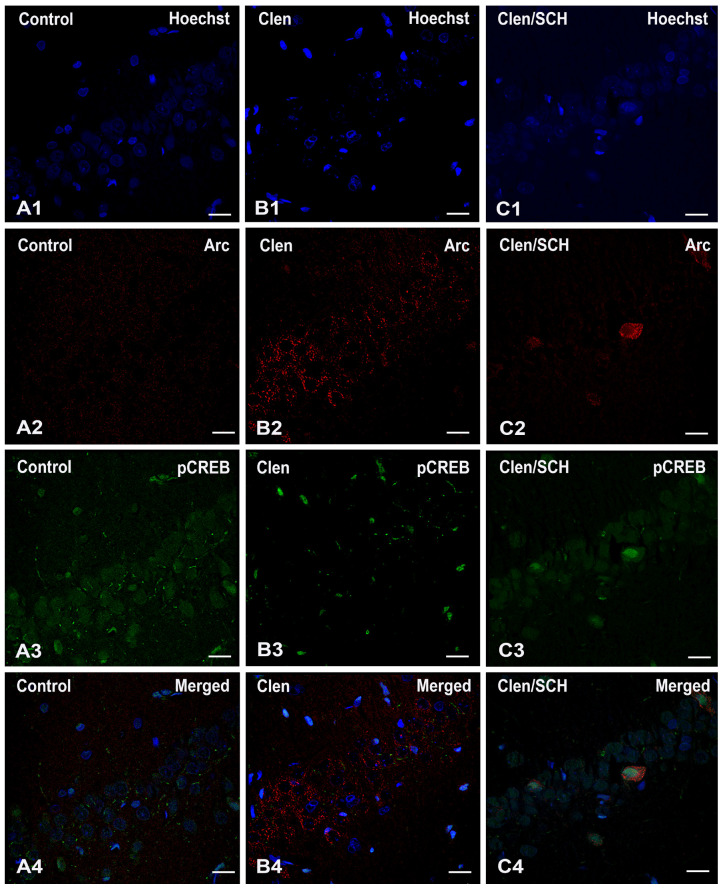
Representative confocal images showing pCREB and Arc in the nucleus of hippocampal pyramidal CA1 neurons following β_2_-adrenergic stimulation and D1/D5 receptor blockade. Double immunofluorescence for Arc (red) and phospho-CREB Ser133 (green), with a Hoechst counterstain (blue), in the hippocampal pyramidal layer under three conditions: (**A1**–**A4**) Control; (**B1**–**B4**) Clen; and (**C1**–**C4**) SCH/Clen. Clen treatment produces a marked perisomatic/perineuronal Arc pattern (**B2**), whereas co-treatment with SCH attenuates the Clen-evoked Arc signal (**C2**). pCREB predominantly localizes to the nucleus across conditions. The merged panels illustrate cellular co-expression with minimal subcellular colocalization. Scale bars: 20 µm.

Dual immunofluorescence for pCREB (green) and Arc (red), with a Hoechst nuclear counterstain (blue), revealed different labelling patterns depending on the condition across the three experimental groups (see Figure 6). The density and distribution of Hoechst-positive nuclei were similar in all groups, indicating no significant cell loss or major changes in brain structure. In the control tissues (Figure 6(A1–A4), panels), Arc staining (Figure 6(A2)) was minimal, and only faint puncta were visible in the neuropil. pCREB also exhibited a weak-to-moderate nuclear signal in a few cells (Figure 6(A3)). Following Clen treatment (Figure 6(B1–B4) panels), Arc localization (Figure 6(B2)) was significantly increased; the perisomatic and somatodendritic areas were densely decorated with puncta surrounding Hoechst-positive nuclei, and many neurons appeared strongly induced for Arc. Local infusion of the β_2_-adrenergic agonist Clen in the BLA selectively enhanced Arc expression in the CA3 (CA3a/CA3b) and CA1 regions, and this effect required D1/D5 receptor activation. In the case of SCH/Clen (Figure 6(C1–C4) panels), the extent of Arc disappearance (Figure 6(C2)) was obvious, being visible only in a few isolated cells or small clusters. A comparison of these pictures at a cellular level reveals co-expression (nuclear pCREB-positive neurons are usually found within Arc-positive perisomatic fields), but very little actual subcellular colocalization. Arc is mainly cytoplasmic/perisomatic or neuritic, while pCREB is nuclear. Overall, the results show that Clen is a major factor in the upregulation of Arc. The co-treatment with SCH, which blunts the Clen-evoked Arc response, does not cause an increase in nuclear pCREB. Rather, SCH decreases pCREB to control levels in most subfields.

### 2.2. The Infusion of Clen into the BLA Did Not Affect Long-Term Memory in Non-Habituated Rats Subjected to the OL Test

**Figure 7:**
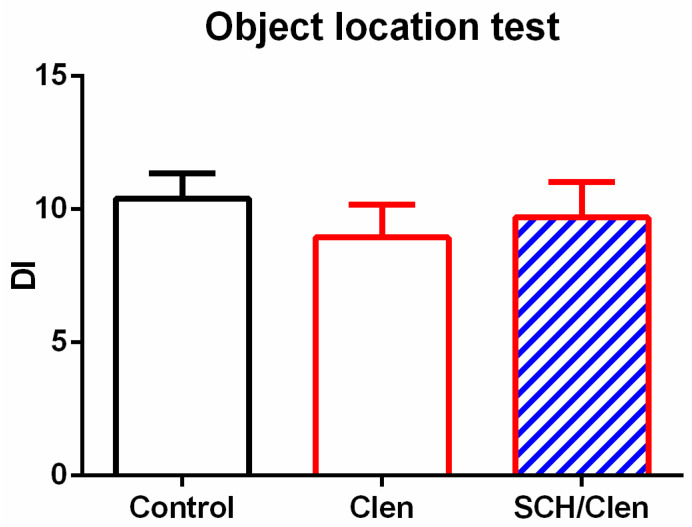
The discrimination index (DI) of Control (white bars) (n = 8), Clen-treated (red bars) (n = 9), and SCH/Clen co-treated (red bars with blue diagonal stripes) (n = 9) groups in the object location test. No significant difference was observed among groups (one-way ANOVA: F2,25 = 0.365, *p* = 0.7).

Of the 30 rats initially assigned to three experimental groups, 26 were included in the object location (OL) test. Four animals were excluded from the analysis because they did not meet the predefined criterion for minimum object exploration (see Section 4, Materials and Methods). The (OL) test was performed twenty-four hours after the training phase. One-way ANOVA analysis demonstrated no significant difference among the three groups (*p* > 0.05) (Figure 7).

## 3. Discussion

Our first experiment provides novel evidence for a probable interaction between dHipp dopaminergic neurotransmission and noradrenaline-dependent activation of the BLA in the context of emotional salience and novelty-induced arousal. Using immunohistological and immunofluorescent methods, we examined the anatomical distribution of Arc and pCREB expression in the dHipp following BLA stimulation during the training session of the OL task and after SCH 23390 administration before Clen infusion.

Several previous studies have reported an increase in synaptic Arc levels in the dHipp after pharmacological stimulation of the BLA via direct infusion of the selective beta-adrenergic agonist, Clen immediately after training sessions for different behavioral protocols [16,20,21,22]. However, to our knowledge, the present work has demonstrated for the first time that the enhancing effect of the post-encoding noradrenergic stimulation of BLA on Arc expression in the dHipp is reduced when the infusion of Clen was preceded by an intraperitoneal injection of the selective D1/D5 antagonist, SCH 23390 suggesting that salient environmental conditions influence neuroplasticity and memory formation in dHipp by activating multiple intertwined modulatory systems, and DAergic neurotransmission, in particular. This notion is further supported by the lack of a substantial effect of the brief low-arousal OL training session on Arc expression in the CA3, CA2, and CA1 areas of dHipp in the Control group. Based on the results of our first experiment, it appears that the interaction between noradrenergic neurotransmission in the BLA and the dopaminergic neurotransmission in dHipp is anatomically restricted, as the injection of SCH 23390 had a more profound effect on the number of Arc positive cells in the medial (CA3b) and distal (CA3a) parts of hippocampal CA3 area, as well as, areas CA2 and CA1 compared to the GrDG and the proximal part of CA3 (CA3c). Since our study does not specify whether Arc expression arises from synaptic origins or from its molecular interactions, we are currently unable to provide a more coherent view of how this difference in the protein’s anatomical distribution relates to experience-induced synaptic plasticity. Nevertheless, the results indicate that during novel or salient situations, the interaction between different direct and indirect neuromodulatory inputs to dHipp influences the molecular mechanism of information storage heterogeneously. Notably, blockade of the D1/D5 receptors also partially reduced the number of neurons positive for pCREB in some areas of the dHipp. Previously, it has been shown in rats that pCREB levels are upregulated in the hippocampus after brief exposure to a novel environment [23]. However, the anatomical dimension of this effect was not reported. Here, we have demonstrated that post-training noradrenergic stimulation of the BLA increases novelty-driven CREB phosphorylation in the GrDG, CA2, and CA1 areas and that, in these same dHipp regions, CREB activation is also dependent on intrahippocampal dopaminergic transmission.

Much to our surprise, during the OL test session, we did not observe enhanced recognition of the object in a novel location in the Clen group, as we had initially expected. The lack of significant results from our second experiment can be interpreted in light of several factors that could influence exploratory behavior in laboratory rodents. For example, animals in the current study did not undergo the preceding habituation session, which is a standard part of the protocol for most object novelty memory tests [24]. During these sessions, animals become familiar with the spatial characteristics of the experimental settings, reducing anxiety and increasing exploration. However, following the evidence provided by McReynolds et al. [21], who found that post-encoding Clen stimulation of the BLA enhances novel object recognition memory and upregulates synaptic Arc in the dHipp only in non-habituated animals, habituation sessions were not conducted. Another possibility is that the close temporal proximity of the pharmacological treatment could also have increased anxiety. In the near future, employing distinct behavioral protocols and further optimizing the timing of our pharmacological interventions would enable us to better assess the potential effects of the interaction between BLA and hippocampal dopaminergic transmission on initial memory consolidation.

It is important to note that our study is not the first one to report an interaction between catecholaminergic neurotransmission and BLA activation in fostering plasticity-related processes. Rodriguez-Duran and colleagues [25] demonstrated in mice that the synergistic optogenetic activation of the glutamatergic BLA and the dopaminergic VTA terminals in the insular cortex induces slow-onset LTP. This effect was not observed when SCH 23390 or the NMDA receptor antagonist APV was administered. Considering dHipp, however, BLA has a purely modulatory role and may reinforce learning-initiated changes in synaptic strength [11,19,26]. This is evident in the study by Frey et al. [26], in which freely moving Wistar rats, electrophysiological stimulation of the BLA 5 or 15 min before and after weak tetanization of the DG promoted the conversion of early-LTP into its long-lasting protein-dependent form. Remarkably, icv administration of propranolol, but not SCH 23390, after LTP induction and 10 min before BLA stimulation, attenuated the observed reinforcing effect. This further endorses the notion that noradrenaline neurotransmission may have a privileged role in modulating plasticity-related processes in the DG [13]. As the BLA does not project directly to the DG, the authors assumed that effects on synaptic plasticity in the DG may result from an interaction between the BLA itself and the direct noradrenergic projection [26]. A subsequent study by Berdago et al. [27] demonstrated that lidocaine injection into the LC abolishes LTP reinforcement induced by BLA stimulation. Furthermore, application of the muscarinic antagonist atropine in LC had the same effect, suggesting that the BLA may further stimulate LC via indirect cholinergic projections. Based on these results, the authors proposed that the BLA and LC, together with other structures such as the main cholinergic nucleus and the medial septum, are part of a larger neural reinforcing system that could enhance ongoing synaptic modification in the DG and determine the course of information storage in natural conditions. Even if our study does not directly address the processes involved in LTP modulation and reinforcement, the reported results regarding the anatomical distribution of Arc complement some of these previous reports, especially if it is assumed that protein expression is a consequence of emerging alterations in synaptic communication and is required to support structural changes in the post-synaptic side. Furthermore, based on the effects on Arc expression that we reveal here, it may be hypothesized that BLA’s impact on ongoing plasticity changes and memory consolidation is achieved in a regionally specific manner through indirect interactions with other modulatory systems that project directly to the dHipp. Notably, in the case of the hippocampal proper, in line with our findings, it could be inferred that noradrenergic activation of the BLA, triggered by a surprising encounter with an emotional stimulus or the general uncertainty associated with novelty, augments dopamine release from axonal terminals in dHipp.

Identifying the physiological mechanism of a potential interaction between the BLA and dopaminergic pathways that reach dHipp is beyond the scope of our current research. Nevertheless, two testable predictions could be outlined for future investigation. Similar to what is proposed in the article of Bergado and colleagues [27], when the BLA is activated during arousing situations, it may become part of a circuit that sustains dopamine release in dHipp, either through direct projection to LC or VTA or through another relay structure that could further stimulate the dopaminergic neuronal populations in these structures. Alternatively, the BLA may influence dopamine release by boosting inputs to dHipp from other memory-related structures, such as the entorhinal cortex (EC). Sonneborn and Greene [28] provided evidence that genetic deletion of presynaptic NMDA receptors located on LC axonal terminals interferes with dopamine-mediated late-LTP in the CA1 area. The authors suggested that enhanced glutamatergic transmission in response to salient stimuli could lead to glutamate overflow binding presynaptic NMDA receptors on LC terminals, thereby promoting dopamine release in the dHipp. On the other hand, Wahlstrom et al. [29] demonstrated that post-training optogenetic stimulation of BLA projections to the medial EC enhances spatial learning and increases Arc expression in the dHipp of male rats. While there is no direct connection between these two studies, we believe that investigating the possible role of BLA as a modulatory controller of dHipp, manifested by amplification of EC to dHipp projections and glutamatergic influence on intrahippocampal dopamine release, is an intriguing research opportunity.

Considering the results of our first experiment in more depth, we found that systemic injection of SCH 23390 did not significantly affect Arc-positive cell levels in the DG, confirming the observations of Frey and colleagues [26] that dopamine is not essential for BLA-mediated effects on plasticity in this region. Surprisingly, there was no significant difference in the number of Arc-positive cells in the DG between the Control group and animals that underwent only a Clen infusion in the BLA. As the animals in this experiment were sacrificed 60 min after pharmacological treatments, we cannot exclude the possibility that BLA stimulation has a long-lasting effect on Arc production in dHipp that could be exhibited either through a change in the dynamics of a single expression wave that could reach its peak at a different time point or through multiple subsequent waves of expression. Ramirez-Amaya and colleagues [30] showed that, unlike in other hippocampal fields, in the upper blade of the DG in rats, two exploratory sessions in a novel environment elicited a single expression wave that lasted up to 8 h. To obtain a more robust understanding of the effect of adrenaline-dependent BLA stimulation in the DG, it will be interesting to examine Arc expression at multiple time points, with and without a preceding exposure to novelty.

Currently, there is a lack of substantial evidence regarding dopamine’s effects on plasticity or the expression of plasticity-related proteins in the CA3 area [31,32]. Nevertheless, Wagatsuma et al. [33] have shown that dopamine released from LC terminals in CA3, but not in DG or CA1, is indispensable for encoding a memory of a novel spatial environment in mice. Concomitantly, there are no detailed reports describing the effects of pharmacological or electrophysiological stimulation of the BLA on dorsal CA3 plasticity. Still, a study by Bass and Manns [34] demonstrated that brief electrical stimulation of the BLA enhances low-gamma frequency-dependent synchrony between CA3 and CA1, thereby positively influencing object recognition memory. The CA3 region is anatomically diverse along its proximodistal axis—the entorhinal input to the proximal subdivision (CA3c) is lighter compared to the medial (CA3b) and distal (CA3a) subdivisions, while projections originating from the DG are denser to the proximal side, but lessen towards the distal end [35,36]. The distribution of Arc in dorsal CA3 reported by the present study highlights the heterogeneous nature of this hippocampal subfield, with post-encoding Clen infusion in BLA profoundly increasing the number of Arc-positive cells in CA3b and CA3a, which was significantly reduced in the SCH 23390/Clen group. We did not observe a significant effect of SCH 23390 on Arc expression in CA3c, allowing us to assume that, as in DG, any hypothetical BLA-mediated impact on the expression of plasticity-related proteins in this microcircuit may be mediated through an interaction with a different neuromodulatory agent. Since CA3b and CA3a receive greater projections from EC, these results can be related to one of the hypothetical frameworks outlined above, as Clen-dependent BLA stimulation could have amplified EC to CA3 input, prompting dopamine release through extracellular glutamate overflow.

Another intriguing aspect of our results is the moderate but notable increase in Arc-positive cells in area CA2 of dHipp in BLA-stimulated animals compared with the Control group. Just as with CA3b and CA3a, SCH 23390 decreased this effect. Area CA2 is a hippocampal field that receives diverse modulatory inputs and plays a significant role in processing social information and in responding to changes in the surrounding environment [37,38]. Recently, Chua and colleagues [39] discovered that activation of dopaminergic D1/D5 receptors in CA2 enhances protein-dependent late-LTP. Thus, our results underscore the need to further elucidate how alterations in dopaminergic neurotransmission triggered by salient stimuli govern experience-driven neuroplasticity in this region.

The effect of SCH 23390 injection on Arc expression in area CA1 is not surprising, given numerous studies demonstrating the importance of dopamine for synaptic plasticity, initial memory consolidation, and memory linking in this area [10,40,41]. In their study, Fuchsenberge et al. [42] found that dopamine in hippocampal slices increases protein synthesis required for LTP, specifically the production of the GluA1 subunit of AMPA receptors. Our in vivo experiment supports the notion that dopamine induces plasticity-related protein synthesis in CA1, especially after novel and arousing events, and further demonstrates that this effect may require an additional amplifying signal, which, in this case, was provided by BLA activation. Finally, systemic injection of SCH 23390 heterogeneously influenced CREB phosphorylation, with the most prominent effect in CA2 and CA1 areas. The selective D1/D5 receptor antagonist SCH 23390 also affected pCREB in GrDG, where the number of pCREB-positive cells was exceptionally high across all groups. The activation of different intracellular signaling cascades by glutamate, growth factors, and various other neurotransmitters could trigger pCREB [43,44], which may explain why the effect of the D1/D5 antagonist did not spread more widely. As a transcription factor, CREB is essential for neuronal excitability and for regulating the expression of genes encoding plasticity-related proteins and memory consolidation [43,44,45]. The apparent decrease in pCREB-positive cells in the dorsal GrDG caused by SCH 23390 indicates that dopamine, even if not indispensable for LTP reinforcement and the synthesis of plasticity-related proteins in the region, could nevertheless influence gene expression important for information storage. Conversely, in the dorsal CA1 area, the interaction between dopaminergic neurotransmission and CREB activation may be particularly important for synaptic plasticity, since it has been demonstrated in acute slices from a transgenic mouse line that functional blockade of the CREB family of transcriptional factors interferes with dopamine-dependent reinforcement of LTP [46]. Besides dopamine-dependent effects on CREB phosphorylation, the elevated number of pCREB-positive cells in DG, CA2, and CA1 in rats from the Clen group suggests that post-training noradrenergic stimulation of BLA increases the general excitability of the neurons in these regions. Hypothetically, this could be interpreted as additional evidence for the role of the BLA in amplifying EC input in dHipp, but this possibility remains to be tested.

### Limitation of the Study

It is important to note again that while the current research provides a good starting point for a more thorough future investigation of a plausible interaction between BLA activation and intrahippocampal dopaminergic neurotransmission in arousal-driven memory modulation, our study is purely exploratory in nature, and the pharmacological methods that we utilized could not provide additional evidence for the physiological mechanism or the exact anatomical organization of such interaction. Our choice of Arc as a marker for this possible connection was guided entirely by previous research on the emotional modulation of memory consolidation and dopamine-mediated effects on hippocampal plasticity. However, since our study does not examine intrahippocampal dopamine levels after clenbuterol-induced BLA stimulation, we cannot exclude the possibility that other factors also influenced Arc expression. With these limitations in mind, we have outlined two hypothetical scenarios to put our results in context and provide guidance for a more sophisticated experimental approach. Testing the validity of our predictions requires implementing methods that allow us to target the individual structures that compose the proposed modulatory circuits. Moreover, tracing how stimulation or inhibition of structures such as the LC and BLA influences dopamine release in dHipp would elucidate their significance in regulating Arc expression, as reported here. The combination of chemogenetic approaches, such as DREADD, and techniques for continuous monitoring of the extracellular molecular composition, such as microdialysis, may be the most promising strategy for addressing the questions our study raises. Alternatively, the application of genetically encoded fluorescent sensors for dopamine and noradrenalin in dHipp and BLA, combined with various behavioral protocols designed to study the effect of novelty or acute stress (open field exploration, social novelty, elevated platform) on memory consolidation, could shed light on the dynamics of catecholaminergic release during active behavior. The simple experimental design of the present study cannot provide a more detailed account, but the results obtained indicate that the connection between BLA and intrahippocampal modulatory inputs during novel and emotionally arousing conditions warrants further attention.

## 4. Materials and Methods

### 4.1. Animals

Young male Wistar rats (1 month old) were purchased from the vivarium at the Institute of Neurobiology, BAS. They were accommodated to a reversed light–dark regime (07:00 a.m. light off\07:00 p.m. light on). A month after arrival, mature rats underwent a surgical procedure to implant cannulas in the BLA. The rats were housed under standard conditions (22–23 °C, in transparent cages, with free access to lab chow (carbohydrates 50%: protein 20%: fat 5%) and tap water).

The experiments were performed in accordance with Council Directive 2010/63/EC on animal experiments and approved by the Bulgarian Food Safety Agency (research project No. 349).

### 4.2. Drugs

The selective β_2_-receptor agonist Clenbuterol hydrochloride (Cat. No. C5423; FOT Ltd., Sofia, Bulgaria) was infused bilaterally into the BLA at a dose of 4 ng/0.5 µL over 60 s. For the infusion procedure, a 30-G injection cannula, connected to a 1 µL Hamilton syringe via a polyethylene tube, was protruded 2 mm beyond the tip of the lead cannula. After infusion, the needle was left in place for at least 60 s. The selective D1/D5 receptor antagonist SCH 23390 hydrochloride (Cat. No. D054; FOT Ltd., Bulgaria) was injected subcutaneously (s.c.) at a dose of 0.2 mg/kg. The drugs were dissolved in sterile 0.9% saline. In combination, SCH 23390 was administered 15 min before the adrenergic agonist. Drug treatment was conducted immediately after the training phase of the OL test. In the control group, the animals received an s.c. injection and infusion of vehicle in the same volume as in the drug-treated groups. All drug solutions were freshly prepared before the experiment. The Clen and SCH doses were based on previous studies [47].

### 4.3. Surgery Procedure and Experimental Design

For *Experiment #1*, the rats were perfused for IMC analysis of pCREB and Arc expression in the dHipp after the training phase in the OL test. For *Experiment #2*, the second cohort of rats was tested 24 h after the training phase in the OL test for memory retrieval. Deeply anesthetized rats in *Experiment #1* and *#2* (ketamine, 80 mg/kg, i.p., and xylazine, 20 mg/kg, s.c.) were fixed on a stereotaxic apparatus (Stoelting Co., Wood Dale, IL, USA) and bilaterally implanted in the BLA with two 22-G cannulas according to the atlas of Paxinos and Watson [48] (coordinates: AP = −2.5 L = ±4.8; H = 6.5) as was described in our previous study [49]. After implantation of the canulae, the rats were randomly assigned to three groups within each cohort before treatment.

### 4.4. Object Location (OL) Test

The OL task was performed in an open field box with black walls (50 cm × 50 cm × 50 cm). Behavioral testing was performed under dim illumination. A measurement of approximately 20 lux was obtained at the arena floor, and the light was distributed evenly across the testing apparatus. A video camera placed above the box recorded both stages of the protocol, and the video recording was used for subsequent analysis. The OL protocol is similar to the one used by Barsegyan et al. [50]. In brief, for the training session, two identical plastic objects (A1 and A2) were placed in front of one of the sidewalls of the OL box close to the corners. During the training sessions, animals were allowed to explore the objects freely for 4 min. The test session was performed 24 h later in the same room. Before the test session, one of the objects was placed close to the center of the OL box (Nov), while the other remained in the same location as in the training session. The test session was also 4 min long. Rats showing abnormal locomotor activity, insufficient object exploration (<10 s total), a higher than 60% preference to one of the two familiar objects in the pre-test, or evident motor impairments were excluded from the analysis. All exclusions were applied blinded to the experimental group. Four out of 30 (1%) rats were excluded due to the reasons previously described. As in our previous study [49], the exploration time (s) was defined as the time from when the nose or forelimbs approached the object. The discrimination index (DI) was calculated as follows: DI = Nov/A1 + Nov. The apparatus and objects were cleaned with alcohol after each trial.

### 4.5. Immunohistochemistry and Immunofluorescence

Animals in all three groups were deeply anesthetized with urethane (1500 mg/kg) and transcardially perfused with 150 mL ice-cold 0.05 M phosphate-buffered saline, followed by 500 mL chilled 4% paraformaldehyde in 0.1 M phosphate buffer (pH 7.4). Brains were removed, post-fixed overnight at 4 °C in the same fixative, embedded in paraffin, and cut into 6 µm coronal sections. Following deparaffinization, immunohistochemical detection of ARC (Arc, ARG3.1; Activity-regulated gene 3.1 protein homolog) and phospho-CREB (Ser133) was performed using the UltraTek HRP Anti-Polyvalent Detection System (AFN600; ScyTek Laboratories, Logan, UT, USA). Antigen retrieval was carried out in a water bath (WB-4MS) at 95 °C for 20 min in citrate buffer (pH 6.0) (ScyTek Laboratories, Logan, UT, USA). After cooling the sections for 20 min at room temperature, they were rinsed in TBST (Tris-buffered saline with 0.05% Tween-20, pH 7.6), endogenous peroxidase was quenched with 3% hydrogen peroxide in distilled water for 10 min at room temperature, and non-specific binding was blocked with Super Block (ScyTek Laboratories, Logan, UT, USA) for 10 min. After three brief TBST washes, biotin blocking was performed with the Biotin blocking kit (ScyTek Laboratories, Logan, UT, USA): 15 min in part A, followed by a wash, then 15 min in part B. All antibody solutions were prepared in ScyTek’s Tris-based primary antibody diluent (ATG125). Sections were incubated overnight at 4 °C with mouse monoclonal anti-ARC (1:1000; Proteintech, Rosemont, IL, USA, 66550-1-Ig) and rabbit polyclonal anti-phospho-CREB (Ser133; 1:1000; Affinity Biosciences, Cincinnati, OH, USA, AF3189). The following day, slides were treated sequentially with the UltraTek biotinylated secondary reagent and HRP-conjugated detection reagent (AFN600), visualized with the DAB Chromogen/Substrate Kit (ScyTek, Logan, UT, USA), counterstained with hematoxylin, dehydrated through graded alcohols, and coverslipped. Negative controls were processed identically except that the primary antibody was replaced with antibody diluent.

Immunofluorescence followed the same workflow, beginning with the Super Block pre-treatment step. Sections were incubated with mouse monoclonal anti-ARC (1:1000; Proteintech, 66550-1-Ig) and rabbit polyclonal anti-phospho-CREB (Ser133; 1:1000; Affinity Biosciences, AF3189). Nuclear DNA was labeled with Hoechst 33342, ultra-pure grade (CAS 23491-52-3; 1:1000; Santa Cruz Biotechnology Inc., Dallas, TX, USA). After washing, goat anti-rabbit IgG (H + L) AF488 (1:100; Elabscience, Houston, TX, USA, E-AB-1055) and goat anti-mouse IgG (H + L) AF594 (1:100; Elabscience, E-AB-1059) were applied for 1 h at room temperature in the dark. Slides were mounted with FluoreGuard Mounting Medium (Hard Set; FMH-060; ScyTek Laboratories, Logan, UT, USA) and imaged on a Leica TCS SPE fluorescence microscope using LAS X software (v3.5.7.23225; Leica Microsystems, Wetzlar, Germany).

### 4.6. Photodocumentation and Image Analysis

Following immunohistochemical staining for Arc and pCREB, slides were examined and imaged using a Leica DM1000 bright-field microscope equipped with a Leica DFC 290 digital camera. Illumination and acquisition parameters were kept constant for all images. Files were saved in TIFF format and minimally processed in Adobe Photoshop CS5 (v12.x; Adobe Systems Inc., San Jose, CA, USA) to remove artifacts.

For cell-density quantification, section images and regions of interest were digitized and analyzed in ImageJ (version 1.54, National Institutes of Health, Bethesda, MD, USA). Immunopositive cells were counted with the Cell Counter plugin (v2.22; K. De Vos). Areas of interest were delineated manually, and counting was implemented by an experienced researcher blinded to group allocation. Analyses were carried out on 6 µm coronal paraffin sections of the hippocampus corresponding to −3.12 to −4.68 mm from Bregma, as defined by a standard rat brain stereotaxic atlas of Paxinos and Watson [48].

### 4.7. Statistical Analysis

Experimental data are presented as mean ± S.E.M. One-way ANOVA followed by Tukey’s test or Kruskal–Wallis on ranks followed by the Mann–Whitney U test, depending on data distribution, were used. SigmaStat^®^ (version 11.0, San Jose, CA, USA) and GraphPad Prism 6 software were used for statistical analyses and figure preparation. The significance level was set at *p* < 0.05.

## 5. Conclusions

**Figure 8:**
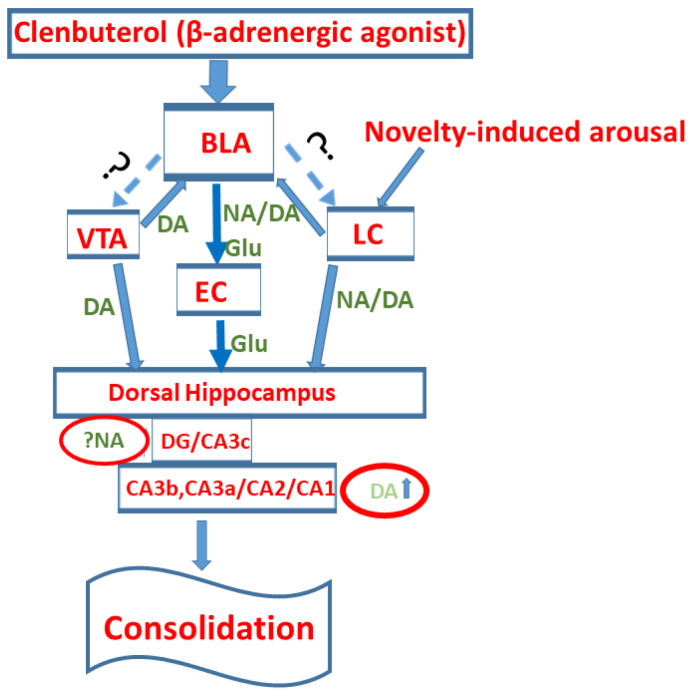
A hypothetical model of region-specific interactions between the basolateral amygdala (BLA) modulatory inputs and intrahippocampal dopaminergic transmission based on the results of the current study. Following a novel or emotionally arousing experience, noradrenaline (NA) and dopamine (DA) are released from the locus coeruleus (LC), activating both the BLA and the dorsal hippocampus (dHipp). Activation of the BLA provides an amplifying signal either through projections to the entorhinal cortex (EC) or recurrent projections to catecholaminergic nuclei (LC and/or ventral tegmental area (VTA)). This signal potentiates dopamine neurotransmission in the dHipp. This BLA-dependent impact on catecholaminergic transmission promotes the region-specific phosphorylation of cAMP response element-binding protein (CREB) and the expression of activity-regulated cytoskeleton-associated protein (Arc) within the dHipp, particularly affecting the dentate gyrus (DG), CA2, and CA1 subregions for pCREB, and the CA3b, CA3c, CA2, and CA1 subregions for Arc. This interaction is considered essential for the initial stages of memory consolidation.

Our findings support the hypothesis that a region-specific interaction exists between BLA modulatory inputs and intrahippocampal dopaminergic transmission, which is considered essential for the initial stages of memory consolidation (Figure 8). This influence occurs through critical interactions with dopaminergic neurotransmission that directly target the dHipp. In our in vivo studies, we found that following a novel or salient experience, dopamine effectively drives the synthesis of plasticity-related proteins in the distal CA3 and CA1 regions. This process appears to depend on an additional amplifying signal from BLA activation, highlighting the intricate relationship between these neural circuits. Following training, noradrenergic stimulation of the BLA elicited significant novelty-driven CREB phosphorylation in the DG, CA2, and CA1 regions. Moving forward, implementing distinct behavioral protocols—with and without prior novelty exposure—coupled with optimizing the timing of our pharmacological intervention, will allow us to thoroughly evaluate the profound effects of the interaction between the BLA and dopaminergic transmission in the dHipp on the initial stages of memory consolidation.

## Data Availability

The original contributions presented in this study are included in the article. Further inquiries can be directed to the corresponding author.

## Abbreviations

The following abbreviations are used in this manuscript:

Arc	activity-regulated cytoskeleton-associated protein
BLA	basolateral amygdala
Clen	clenbuterol
DG	dentate gyrus
EC	entorhinal cortex
dHipp	dorsal hippocampus
GrDG	granular layer of the DG
LC	locus coeruleus
long-LTP	long-lasting form
LTP	long-term potentiation
OL	object location
pCREB	phosphorylated form of cAMP-response element-binding protein
s.c.	subcutaneously
VTA	ventral tegmental area
